# The 16th international oncolytic virotherapy conference: from *in vitro* to clinical studies, novel approaches in cancer therapy meeting report

**DOI:** 10.3389/fimmu.2025.1554767

**Published:** 2025-03-26

**Authors:** Elena Ekrami, Parisa Ghanbari, Eman M. Othman, Aladar A. Szalay

**Affiliations:** ^1^ Cancer Therapy Research Center (CTRC), Department of Biochemistry-I, Biocenter, University of Wuerzburg, Wuerzburg, Germany; ^2^ Department of Pathology, Center for Clinical Science Research, Stanford School of Medicine, Stanford, CA, United States

**Keywords:** 16 th international oncolytic virotherapy conference, immunotherapy, *in vitro* studies, clinical studies, cancer therapy

## Abstract

The 16^th^ International Oncolytic Virotherapy Conference, which was held in the vibrant city of Rotterdam, Netherlands, on 27-30^th^ November 2024, featured a variety of novel strategies for improving oncolytic virus therapy. A reflective keynote highlighted industry challenges and lessons. Presentations covered the cutting-edge developments in oncolytic virotherapy from scientific, clinical, and industry perspectives. Young Investigators Talk was a newly introduced segment that debuted this year.

## Introduction

**Figure 1:**
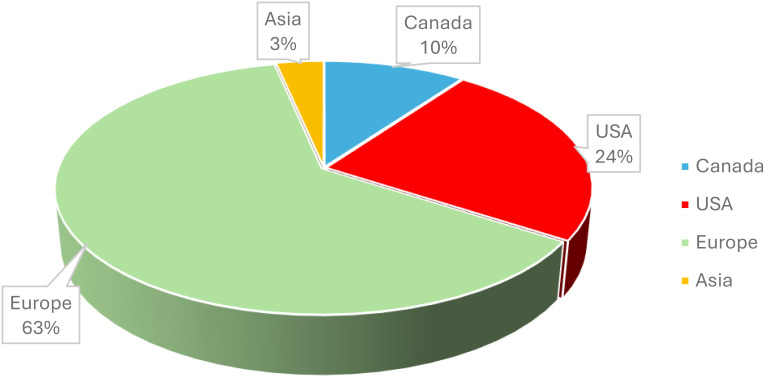
An overview of the participating institutions and clinics: worldwide distribution.

This conference, which began in 1997 with colleagues working on early development of oncolytic viruses (OVs), has developed into the oldest and the most authoritative international conference on OV therapy. From Sunday, 27 until Wednesday, 30 October 2024, OV researchers from around the world (**Table 1**, **Figure 1**) convened in beautiful Rotterdam, Netherlands, for the 16th annual International Oncolytic Virotherapy Conference (IOVC). This conference featured renowned speakers from around the world, providing opportunities for networking, engaging in social events, attending scientific presentations, participating in poster sessions, and much more. We are grateful to the organizers for creating an excellent program and conference, including Dr. Martine Lamfers (1), Associate Professor at Erasmus MC, and Dr. Clemens Dirven, Symposium Chair, at Erasmus MC and the IOVC international organizing committee.

**Table 1:** An overview of the participating institutions and clinics: worldwide distribution.

Canada	USA	Europe	Asia
Virogin Biotech	Mayo Clinic	StemVac GmbH, Germany	Osaka University Hospital, Japan
Oncolytics Biotech	Candel Therapeutics	TILT Biotherapeutics, Finland.	Tottori University, Japan
Children’s Hospital Research Institute	Memorial Sloan Kettering Cancer Center	ORCA Therapeutics, Netherlands	Weizmann Institute, Israel
University of Sherbrooke	Calidi Biotherapeutics	Francis Crick Institute, UK	Chongquing University, China
University of Ottawa	MD Anderson Cancer Center		
Ottawa Hospital Research	Vyriad	ViraTherapeutics GmbH, Germany	
University of Calgary	Replimune	Leeds Institute of Medical Research, UK	
		University of Leeds, UK	
		University of Oxford, UK	
		The Institute for Cancer Research, UK	
		University of Sheffield, UK	
McMaster University	Brigham &Women’s Hospital	Umea University, Sweden	
CHEO Research Institute	Duke University	German Cancer Research Center, Germany	
		Technical University Munich, Germany	
		Virotherapy Center Tubingen, Germany	
		University of Tubingen, Germany	
		University Hospital Tubingen, Germany	
		Witten/Herdecke University, Germany	
		University of Wurzburg, Germany	
		Abalos Therapuetics, Germany	
	Mayo Clinic	Universite de Paris, France	
		Oncovita-Institut Pasteru, France	
		Transgene S.A., France	
		CNRS, Paris, France	
		Institut Gustave Roussy, France	
	Oncolytics Biotech	KU Leuven, Belgium	
		University of Liege, Belgium	
		Exothera Jurnel, Belgium	
	Ubrigene Biosciences Inc	University of Bologna, Italy	
	University of Illinois	University of Helsinki, Finland	
	Icahn School of Medicine at Mount Sinai	Leiden University Medical Centre, Netherlands	
		Erasmus Medical Center, Netherlands	
	University of Massachusetts	Wageningen Bioveterinary Research, Netherlands	
		Medical University Innsbruck, Austria	
	University of California	University of Zagreb, Croatia	
	University of Minnesota	Hansa Biopharma AB, Sweden	
	Rochester Institute of Technology	Maria Sklodowka-Curie National Institute of Oncology, Poland	
	University of North Carolina	Aarhus University, Denmark	

The meeting began with a welcome address by Dr. Martine Lamfers (Erasmus MC, Netherlands), followed by a presentation titled ‘Rotterdam, the City of Dreamers and Doers’ by Catherine Kalamidas (Rotterdam Partners). The first keynote was presented by Prof. Dr. Guido Kroemer (Université de Paris, Sorbonne Université & Institute Gustave Roussy, France) on immunogenic cell death (ICD) in cancer therapy. In this topic, Kroemer introduced Talimogene Laherparepvec (T-VEC) (a derivative of Herpes Simplex Virus) as a perfect recipe for stimulating immunogenicity. Data showed that combining OVs with existing ICD-promoting therapies (chemotherapy, immunotherapy) maximizes tumor eradication. For identifying ICD inducers, they offered a pipeline: specifying a library of compounds or genes, screening for ICD hallmarks on biosensor cell lines, and *in vitro* and *in vivo* validation. Results indicated that ICD enhancers play a pivotal role in amplifying immune response by activating Conventional type 1 dendritic cells (cDC1) and inducing Type I IFN response. Additionally, they ensure a sustained immune response in tumors.

The second keynote was presented by Prof. Abishek Garg (KU Leuven, Belgium) on immunotherapy resistance in tumors, exploring the complex interplay between immune cells and the tumor microenvironment (TME), emphasizing insights from his cutting-edge research. He highlighted the distinctions in immune landscapes across tumor types, which revealed the superior tumor-reactive CD8+ T cell responses in microsatellite instability (MSI) vs. microsatellite stability (MMS) colorectal cancers. Garg demonstrated the identification of hypofunctional T cell states impacting therapeutic outcomes, especially in Glioblastoma (GBM). The spatial dynamics of immune cells were further elaborated, where a spatially embedded HLA signature predicted immunotherapy success in advanced renal cell carcinoma by examining interactions between tumor-associated macrophages (TAMs) and pre-exhausted CD8+ T cells. Garg’s talk provided a comprehensive framework for addressing immunotherapy resistance through both spatial and functional immune profiling, opening avenues for personalized cancer treatment. Both keynote presentations were thought-provoking and challenging (2–5).

As the symposium progressed, the presentation of promising preclinical and clinical results rekindled interest by demonstrating the efficacy of OVs in systemic administration as well as across a range of cancer types. The insightful keynote speech set the stage for a comprehensive exploration of the latest advancements in OV therapy.

## Pre-clinical achievements

The scientific community has acknowledged oncolytic virotherapy combined with immunotherapy as a promising therapeutic and perhaps curative strategy for a variety of tumor types. At the conference, one of the most important discussed topics was the pre-clinical findings related to the mechanisms of oncolytic virotherapy and related immunotherapy, tumor microenvironment, biomarkers of response and the recent and novel platforms and payloads, in addition then investigators emphasized the role and potential of the combination therapies.

### Mechanism of action

Several investigators reported the mechanisms in which OVs activate anticancer immunity. A key highlight of this segment was the presentation delivered by the invited speaker; Dr. Richard Vile (Mayo Clinic, USA). To enhance anti-tumor therapy, they engineered Oncolytic Simian Foamy Virus (oFV) to display the CD19 antigen, a CAR T cell target, hypothesizing that this would promote CAR T-mediated tumor clearance in cells resistant to oncolysis. In subcutaneous tumor models, oFV-CD19 demonstrated persistent intratumoral infection with modest anti-tumor effects, while adding anti-CD19 CAR T cells improved clearance of infected tumor cells. However, the most effective tumor clearance occurred with high-dose oFV alone, as CAR T cells eliminated infected cells, reducing the viral spread necessary for sustained therapy. Escaped tumors revealed deletions in the oFV-CD19 transgene, allowing the virus to evade CAR T clearance. These findings also highlighted the potential of oFV as a slow-spreading oncolytic virus, suggesting future exploration of non-clearative immune-stimulatory strategies to condition tumors for effective immunotherapy (6). Dr. Jennifer Altomonte (Technical University of Munich, Germany) showed the administration of Vesicular stomatitis virus (VSV)-Newcastle disease virus (NDV), whether given intratumorally or intravenously, led to an early influx of NK cells, T cells, and dendritic cells (DCs) in both the TME and systemically. It also induced the expansion of both tumor antigen-specific CD8+ and CD4+ T cells within the respective lymph node and tumor. The depletion of CD8+ T cells restored the therapeutic effects, as their presence was crucial to the ability of the virus to work. Moreover, delivery of high-affinity soluble PD-1 by VSV-NDV greatly amplified the immune response (elevated CD8+ T cells and extended life span) in a hepatocellular carcinoma (HCC) mouse model. Results provided evidence for VSV-NDV as a possible solid tumor immunotherapy and a suggestion that it may also be synergistic with other forms of immunotherapy (7).

Dr. Nitya Mohan (London Institute of Cancer Research, UK) presented another study on the interaction of an oncolytic herpes simplex virus (RP1, Replimune Inc.) with the immune system in head and neck cancer and melanoma. RP1 uniquely propagated in human head and neck squamous cell carcinoma (HNSCC) cell lines, but not in immune cells such as monocytes or T cells, when examined using *in vitro* assays. RP1 exposure to tumor cells induced superior priming of tumor-associated antigen (TAA)-specific cytotoxic T lymphocytes (CTLs) against HPV E7 and MAGE-A1. Importantly, the addition of a fusogenic protein (GALV-GP R-) was found to enhance anti-tumor CTL responses in RP1. Results illustrated the ability of RP1 to enhance immune activation following its infection of tumor cells and thus hold promise for better oncolytic virus therapies in cancer.

Dr. Michael Brown (Duke University, USA) also investigated the relationship between innate inflammation pre-treatment and post-PVSRIPO (recombinant poliovirus) survival for GBM patients and approached modulating this parameter. He showed that increased pre-treatment intratumoral neutrophil density, MHC-class II expression, and inflammatory cytokines in the blood correlated with improved survival after PVSRIPO. In addition, they found that pre-treatment levels of IL-1b and type I interferon responses posttreatment were predictive of treatment outcome. In addition, peripheral immunization for non-tumor antigens amplified global inflammation and improved tumor bearers’ response to PVSRIPO and STING agonist treatment in glioma models. In sum, results indicated that the efficacy of glioma virotherapy could be improved by targeting innate immunity. Other studies investigated deciphering translation control in GBM cells during Maraba virus MG1 oncolysis (8). Aida Said (University of Ottawa, Canada) indicated that infection by MG1 disrupted transcription and translation, causing the two processes to decouple. Using RNA-seq and Ribo-seq, the team found a purine-rich motif in the 5′ UTRs of genes that were highly translationally active, including GLDC, and enhanced translation when overall protein synthesis was inhibited by MG1. This motif was further validated through luciferase assays whereby translation suppression by MG1 could be alleviated. Furthermore, polysome profiling also confirmed that GLDC translation was increased while its mRNA levels were low, so targeting control of translation may be an avenue for improvement in oncolytic virus therapy.

These studies shed light on the complex ways OVs boost anticancer immunity, revealing their exciting potential as therapies. They showed how OVs can trigger strong T-cell responses, transform the TME, harness innate immune defenses, and influence protein synthesis control. Together, these findings highlight the diverse and powerful strategies OVs use to modulate the immune system in the fight against cancer.

### Tumor microenvironment

To improve the oncolytic capacity of viruses and the anticancer immune responses, some groups have focused on the TME. Dr. David Johnson (University of Oxford, UK) and his group used resected liver lobes with tumors from cancer patients to test therapies in conditions that mimic human physiology. They connected three resected liver lobes containing tumors (cholangiocarcinoma or metastatic colorectal cancer) to the Metra^®^ machine, and an Adenovirus serotype-11(Ad11)-based “traffic light” virus (TLV) was introduced into the liver lobes. They were kept functional for up to 60 hours after dosing, and tissue samples were collected and analyzed. Results showed that tumor cells demonstrated active viral replication at later stages, with normal cells showing only early signs of infection. They offered this method as an efficient tool for studying liver tumors and testing oncolytic virus therapies in comparison to animal models. Another study at the University of Oxford, UK, presented by Dr. Ahmet Hazini, aimed to develop an anti-CD40-anti-EpCAM bispecific molecule (BiMEs) that connects antigen-presenting cells (APCs) and cancer cells to improve antigen uptake and presentation by APCs. Researchers developed an oncolytic adenovirus encoding the anti-CD40-anti-EpCAM BiME (9).

Fresh human colorectal tumor and normal colon biopsy samples were infected with the developed oncolytic adenovirus to test its replication and BiME production. Results showed BiME significantly enhanced the phagocytosis of cancer cells by macrophages, and it induces antigen cross-presentation. Based on their research, oncolytic adenoviruses (OAds) encoding BiMEs provide a new mechanism for antigen-agnostic cross-presentation, improving immune responses against cancer for developing new cancer vaccines targeting solid tumors. Other studies collectively highlighted the versatility of OVs in reprogramming the TME, modulating immune responses, and enhancing combination therapies. Dr. Liang Deng (Memorial Sloan Kettering Cancer Center, USA) and his colleagues created Interferon Alpha and Beta Receptor Subunit 1 (IFNAR1) conditional knockout mice using a Cre-IoxP system, which allowed for examining the role of IFNAR signaling in myeloid and T cells for vaccinia virus-induced antitumor response. Results demonstrated that both IFN production and type I IFN receptor signalling are required for antitumor effects triggered by viruses. Significantly, they also showed that IFNAR signalling on different cell types (macrophages/granulocytes & Tregs/CD4/CD8 T cells) is important for virus-induced antitumor effects. Their findings shed light on the complex interaction of multiple responses of anti-cancer immunity (10). Another study demonstrated by Dr. Candelaria Gomez-Manzano (MD Anderson Cancer Center, USA) demonstrated that, utilizing stable knockdown cell lines and syngeneic mouse glioma models, how the release of the second messenger Cyclic guanosine monophosphate-adenosine monophosphate (cGAMP) by brain tumor cells can regulate immune responses to oncolytic virus infection. This interferon-independent cGAMP response acts upstream of the interferon and depends on non-POU domain-containing octamer-binding (NONO) protein expression in tumor cells. This study indicates an immediate need for new types of therapeutic strategies, like oncolytic virotherapy, which aim to address hurdles including the fast clearance of the therapeutic virus and limited development of suitable anti-tumor immune responses (11).

Dr. Victoria Jennings (Leeds Institute of Medical Research, UK) used Ovs to deliver miRNA to TAMs, leading to a targeted anti-tumor phenotype and enhanced cancer immunotherapy efficiency. The group showed that in ovarian cancer cell lines, ORV (oncolytic rhabdovirus) infection leads to the production of it, it may suppress the tumor-promoting activity of TAMs and boost the therapeutic effect of cancer OV therapy. Tumor-derived extracellular vesicles (TDEV) are also taken by myeloid cells like TAMs. Thus, miRNA-armed ORVs provide a novel means to deliver an immunomodulatory miRNA specifically to tumor-associated myeloid cells. Consequently, it may suppress the tumor-promoting activity of TAMs and boost the therapeutic effect of cancer OV therapy (12).

Finally, Priscilla Kinderman (Leiden University Medical Center, Netherlands) reported results on a new VSV-derived sequence-optimized RIG-I agonist designated as M8, which transforms the TME to improve the efficacy of immune checkpoint therapy in solid tumors. M8 increased the efficacy of anti-PD-L1 in murine models for resistant or semi-resistant tumors together with oncolytic reovirus (Reo), but not VSVdelta51 in their study. M8 and Reo both induced interferon-stimulated gene (ISG) expression and modified myeloid cell localization in tumors to yield a more immunogenic TME. While all three agents enhanced CTL infiltration (albeit to a different degree), CTL infiltration was not the primary mediator of successful combination therapy. Results demonstrated that unlike traditional OVs, M8 can bypass tumor resistance to immune checkpoint inhibitors (ICIs), an important feature that furthers its development as a promising candidate in cancer immunotherapy.

By focusing on the TME, reprogramming immune responses, and boosting the effectiveness of combination therapies, these approaches opened the door to more tailored and powerful cancer treatments. Innovative tools like advanced adenoviruses delivering bispecific molecules, miRNA-equipped rhabdoviruses, and enhanced RIG-I agonists such as M8 showcased the adaptability of OVs in tackling tumor resistance and strengthening antitumor immunity.

### Biomarkers of response

Beyond efforts to increase virus replication in the tumor, several researchers attempted to elucidate the relationship between cancer cell infection and response to OV treatment. Prof. Dr. Dr. Christine Engeland (Witten/Herdecke University, Germany) shared an insightful study on the effectiveness of five clinically relevant OVs (adenovirus Ad5-hTERT, T-VEC, measles vaccine strain MV-NIS, Reovirus mutant jin-3 (jin-3) and H1 protoparvovirus (H−1PV)) against pancreatic ductal adenocarcinoma (PDAC) using 14 patient-derived tumor cultures. According to Engelands’ report, tumors responded differently, with 12 out of 14 cultures demonstrating sensitivity to at least one OV. Interestingly, and in contrast to the classical subtype, the basal-like subtype of PDAC exhibited a greater sensitivity to the H-1PV, jin-3, and T-VEC viruses. In the same study, the researchers also identified different biomarkers that can predict OV responses, showing that therapeutic outcomes are influenced by other factors beyond viral entry receptors. For instance, high levels of ISG expression were associated with resistance to the oncolytic measles virus, while low levels of cyclic GMP-AMP synthase (cGAS) correlated with strong responses. Furthermore, combining OVs with a cGAS inhibitor significantly improved cell killing in resistant PDAC cultures, emphasizing the potential of customized combination therapies to overcome OV resistance. The findings establish the foundation for future research into tailored treatment approaches and demonstrate the potential of personalized oncolytic virotherapy for PDAC (13).

In an effort to increase therapy options for this difficult malignancy, Dr. Jonny Hertzog from the German Cancer Research Center (DKFZ) presented another study on PDAC. This study examined the potential of lesser-known viral strains as oncolytic agents. Using a library of 31 adenovirus types, the researchers assessed each virus’s cancer-killing ability, replication characteristics, and immune activation in patient-derived PDAC models. Several alternative adenoviruses demonstrated higher cytotoxicity and particle production compared to the commonly used human adenovirus type 5. Interestingly, in different PDAC patient cultures, some viruses displayed particular potency profiles that correlated with clinically significant PDAC molecular subtypes instead of receptor expression levels. The creation of subtype-targeted oncolytic adenovirus candidates, which included genomic alterations for safety and improved efficacy, was guided by these findings. The study highlights a promising avenue for personalized immunovirotherapy using the diversity of adenoviruses.

One study focused on identifying the T cell epitopes specific to the VSV- glycoprotein (GP) (VSV-GP) OV to enhance immune monitoring during therapy. VSV-GP, which has shown promising antitumor and immune-stimulating effects, is currently in phase I clinical trials, which were presented by Dr. Guido Wollmann (Medical University Innsbruck, Austria). However, OV treatment activates both antitumor and antiviral T cells, complicating the assessment of treatment-specific immune responses. Using the BALB/c mouse model, the researchers applied bioinformatic epitope prediction and ELISpot assays to identify eleven VSV-GP-specific epitopes that significantly activated CD8+ T cells. These results pave the way for improving VSV-GP’s development by offering more effective methods to study and understand how the immune system reacts to the therapy (14). Finally, Eftychia Stavrakaki (Erasmus Medical Center, Netherlands) investigated the potential of tailoring OV therapy for GBM by examining individual tumor responses to two OVs—Delta24-RGD (Oncolytic adenovirus) and rQnestin34.5V1. Using a panel of 24 patient-derived GBM cultures, the researchers measured the half maximal effective concentration (EC50) and analyzed immune responses via an autologous ex vivo co-culture model. The results revealed considerable differences in how tumors responded to OVs, both in their susceptibility to virus-induced cell death and their type I interferon responses. Interestingly, a strong oncolytic effect didn’t always translate to a robust immune response. Instead, the immune activation was more closely tied to prolonged activation of interferon pathway transcription factors in GBM cells and an immune microenvironment dominated by myeloid cells. These findings suggest that successful OV therapy for GBM requires a more tailored approach, considering specific tumor traits like transcription factor activity and immune cell composition, rather than focusing solely on the tumor’s sensitivity to oncolysis (15).

These findings highlight the promising strategies of tailoring oncolytic virotherapy to each patient, improving its effectiveness and tackling resistance. This approach offers new optimism for treating difficult cancers like PDAC and GBM.

### Novel platforms and payloads

In addition to highlighting improvements to the oncolytic activity of OVs, IOVC16 also showcased novel platforms and payloads for enhancing OV-elicited antitumor immunity. First, Dr. Beata Halassy (University of Zagreb, Croatia) gave a presentation on a fascinating personal journey, and an Unconventional Case Study of Neoadjuvant Oncolytic Virotherapy for Recurrent Breast Cancer. The presentation highlighted a remarkable self-experimentation case involving a 50-year-old female virologist, who also happened to be the patient. Diagnosed with locally recurrent, muscle-invasive breast cancer, she underwent multiple intratumoral injections using research-grade virus preparations developed in her own laboratory. Initially, she utilized the Edmonston-Zagreb measles vaccine strain (MeV), followed by the VSV. The treatment led to a simple, non-invasive tumor resection, showcasing the potential of oncolytic virotherapy in advanced cancer cases. The tumor shrunk significantly two months after injections, and the patient remained refractory-free for 45 months post-surgery. The findings highlight the potential of oncolytic virotherapy as a promising treatment strategy (16).

The keynote presentation was delivered by Dr. Stephan Russel (Vyriad, USA), on engineering an oncolytic cytomegalovirus to recruit T cells into the TME and selectively transduce them with an anti-tumor CAR. Dr. Russel outlined a conceptual approach for using cytomegalovirus (CMV) as a delivery system for lentiviral CAR-T cell therapy to target metastatic cancer. CMV has many advantages as an oncolytic virus; it has a large, stable DNA genome with a higher capacity than HSV-1 to accommodate multiple foreign transgenes. Also, it has a unique interaction with the immune system. Up to 10% of the memory T cell compartment in blood is CMV specific, and human cytomegalovirus (HCMV) is one of the most immunogenic pathogens for humans. HCMV has many ways to evade the immune system, including targeting antigen presentation, mimicking cytokines, and antagonizing innate immune responses. Researchers figured out that CMV-derived peptide epitopes, delivered intratumorally, can mobilize pre-existing CMV-specific T cell responses into the TME and promote epitope spreading against TAAs. Researchers also reported a treatment of a patient (long-term GBM) with CMV. The GBM patient demonstrated a five-year progression-free survival following CMV reactivation. Russel demonstrated the process of engineering CAR molecules onto CMV-specific T cells *in vitro*. PBMCs are obtained from a donor or patient. These cells are then stimulated with CMV-specific T cells. T cells are then transduced with a lentiviral vector encoding a dual-specific CAR construct. This approach addresses the key challenges in CAR-T therapy, such as short T cell persistence, limited response duration, and the inability to re-stimulate T cells after relapse or persistent disease. Their previous findings demonstrated that CMV showed potent antitumor activity in an immunocompetent mouse model and enhanced T cell responses in treated tumors. They also found that T cells are involved in oncolytic CMV-induced systemic antitumor immune responses. Results illustrated that CMV vector-encoding CD3-targeted lentivirus for *in vivo* CAR-T cell generation was constructed. CMV vector also enables the conversion of T cells into CAR-T cells and promotes the elimination of cancer cells. These results offer a promising strategy to overcome key limitations in CAR-T therapy.

A new systemic oncolytic virus, EnvRT-01, has been developed by Calidi Biotherapeutics, USA., which was presented by Dr. Duong Nguyen. This virus is designed with a second membrane derived from human cells. The additional membrane protects the virus from immune inactivation, allowing for more efficient systemic delivery of the virus to its tumor target. As a result, EnvRT-01 can reach its tumor target more effectively. They demonstrated that EnvRT-01 (a novel tumor-selective vaccinia virus strain) interfered with human humoral immunity (including neutralizing antibodies) with rapid viral growth in xenograft and syngeneic models. The virus founder strain specifically targeted tumors of lung, melanoma, and head & neck cancers by limiting tumor growth and remodeling the immune speed as a hot microenvironment to advance other immunotherapy strategies. The EnvRT-01 reduced both the sizes of metastatic tumors in the lungs and liver in models of metastasis while increasing lymphocyte numbers (T cells and natural killer cells) within these organs. Thus, this enveloped virotherapy may provide novel means to treat localized as well as metastatic cancers. Dr. Mathieu Crupi (Ottawa Hospital Research Institute, Canada) talked about a new OV-based T-cell engagers (TCE) platform developed for the treatment of cancers through immunotherapy. Engineered vaccinia viruses (VVs) were designed with CRISPR to express bispecific TCEs that would guide the activity of T cells specifically on tumors in preclinical models of solid cancers, including colorectal, breast, and ovarian cancer. These VVs targeted cancer-specific antigens (e.g., CEA, FAP, EGFR), while making the T cells traffic and arming them *in situ* at the tumor. *In vitro* and *in vivo* studies demonstrated enhanced T-cell cytotoxicity against tumor cells, leading to significant tumor reduction. This effect was accompanied by immune responses that prevented tumor regrowth in animal models. The VV-TCE platform in combination with ICIs and other OVs provided improved efficacy, higher levels of T-cell activation, and lower exhaustion markers. Results illustrated the ability of VV-TCEs to enhance tumor-targeted immune responses, which represents a novel approach in cancer treatment (17). Another interesting study presented by Dr. Faith Howard (University of Sheffield, UK) showed an amazing approach to improve systemic oncolytic virotherapy for metastatic disease involving nanoparticle encapsulation. Researchers used biocompatible nanoparticles to encapsulate both enveloped and non-enveloped OV to protect the OV from immune clearance, give efficient systemic delivery of OV to metastatic tumors, which promote tumor infection, reduce tumor burden, and prolong mouse survival in prostate and breast cancer models. Thus, encapsulating the nanoparticles within OV represents a platform that should dramatically enhance treatment of metastatic tumors. In another approach, researchers used engineered Salmonella as vehicles for the delivery of OVs into solid tumors. This study was presented by Shradha Khanduja (University of Massachusetts, USA). In this study, a new strain of Salmonella (BB’CDF) was engineered, which showed high efficiency in delivering the genetic material of oncolytic parvoviruses (MVMp and H1PV) into cancer cells. BB’CDF strain was derived from the parental strain of Salmonella, VNP20009, by knocking out DNA recombination genes using lambda red recombination. Parvovirus stability was evaluated using next generation sequencing. 4T1 cancer cells were incubated with BB’CDF expressing the parvoviral DNA (VDS). 36 hours post infection (hpi), the cancer cells were evaluated for NS1 expression with an immunoblot. In mice with liver cancer, VDS (virus-delivering Salmonella) delivered functional virus and induced improved CD8+ T cell infiltration into tumors, 84% tumor regressions, and increased survival. These results highlighted the potential of Salmonella-based OVs to enhance antitumor immune response, prevent recurrent tumors, and inhibit metastasis, making it a powerful and effective platform for the treatment of solid tumors (18).

Researchers also introduced a new protocol to facilitate the generation of improved oncolytic adenovirus vectors—termed Adenovirus Directed EVOlution (ADEVO). This study, which was presented by Katrin Schröer (Witten/Herdecke University, Germany), reported the use of high-throughput homologous recombination. Ad vectors with random vector variants were created to construct large libraries of adenovirus variants that could be selected for their ability to efficiently infect and lyse various cancer cell lines, including pancreatic carcinoma and other tumor types with low adenovirus permissiveness. Following several rounds of enrichment in GBM, the derived variants showed increased viral replication and greater lysis in all tumor cell lines assessed. The insertion of short peptides into the fiber protein improved antitumor efficacy by increasing the therapeutic properties of adenoviruses. They used these optimized fiber proteins in various adenovirus serotype backgrounds to develop highly efficient oncolytic vectors with enhanced *in vitro* oncolysis. Dr. Schroër offered ADEVO as a novel platform for producing large libraries of varied adenovirus strains with enhanced oncolytic characteristics that could be beneficial for many clinical applications (19).

In another study presented by Dr. Richard Vile from the Mayo Clinic, USA, the researchers have found that the auto-immune regulator (AIRE), a transcription factor in medullary thymic epithelial cells, can mimic T cell immune tolerance by presenting a profile of SELF antigens against which functional T cell reactivity is absent. By altering AIRE expression in tumor cells, these changes can trigger heteroclitic CD8+ T cell-mediated rejection of tumors. High-quality tumor-associated epitopes produced by AIRE enhance this rejection by inducing helper CD4+ T cell responses. With immunotherapies, this strategy can result in successful tumor removal (20). The groundbreaking studies presented at IOVC16 showcase were the fast-paced progress in developing platforms and payloads to improve the systemic delivery and immune-boosting effects of OVs. They highlighted how cutting-edge delivery systems, bioengineering techniques, and evolution-inspired approaches are breaking down barriers in OV therapy, paving the way for exciting new possibilities in cancer treatment.

### Combination therapies

Along with demonstrating enhancements to the oncolytic activity of Ovs, IOVC16 also presented innovative strategies for boosting the antitumor immunity triggered by OVs. Many of these combined OVs with other immunotherapies. Most studies explored how combining OVs with other therapies like immune checkpoint inhibitors (e.g., anti-PD1), CAR T cells, or targeted antibodies (e.g., HER2-targeted TCE) can enhance the effectiveness of oncolytic virotherapy. It has been demonstrated that OV and CAR T cell combination therapy produces TCR-educated CAR T cells (TED-CAR T), which use their T cell receptors to identify viral epitopes. In preclinical models where traditional CAR T therapy failed, TED-CAR T cells significantly cured gliomas and melanoma due to their increased cytotoxicity against tumors, extended *in vivo* persistence, and capacity to be reactivated through OV boosts. Building on this, Dr. Richard Vile (Mayo Clinic, Rochester, USA) presented data supporting the hypothesis that TED-CAR T cells could be generated directly from naïve T cells *in vivo* by using DC vaccines to present viral epitopes while simultaneously producing CAR-encoding retroviral particles. *In vitro* studies demonstrated that peptide-loaded DCs, engineered to produce replication-defective CAR retroviral vectors, could effectively transduce naïve CD4 and CD8 T cells. This procedure produced CAR T cells that were unique to the peptide that the DCs presented as well as the CAR target. When these engineered DCs were transferred into tumor-bearing mice, they led to tumor regression and even cures, with the CAR T cells persisting long-term and being reactivated by viral boosting. These findings show that it is possible to produce a variety of memory and effector TED-CAR T cells *in vivo* from naïve T cells, providing a powerful and long-lasting tumor immunotherapy approach.

Additionally, Dr. Vile provided information on how OVs can enhance CAR T cell phenotype and function in solid tumors by utilizing endogenous TCR signaling. A study was done with vesicular stomatitis virus (VSV) in murine tumor models that showed that this combination creates a unique group of hyper-functional CAR T cells that are primed against viral antigens. These CAR T cells displayed a more persistent and enhanced phenotype (KLRG1hiCD127loCD62Llo). Experiments in C57Bl/6 mice revealed that up to 50% of CAR T cells targeted the VSV N epitope, leading to decreased activation, increased granzyme B and IFNγ production, and a distinctive hyper-effector phenotype. These results indicate that OV-CAR T combination therapy enhances CAR T cell function and endurance, suggesting that incorporating antiviral TCRs into CAR T cells could further improve the efficacy of solid tumor therapy. Also, in another study in Dr. Ville’s group, presented by Benjamin (Luke) Kendall (Mayo Clinic, USA), the investigation focused on how VSV can be used to enhance T cell-mediated antitumor responses. They investigated the interaction between ovalbumin (OVA) and Vesicular Stomatitis Virus (VSV) responses *in vivo* using syngeneic B16 and MC38 tumor models. CD8 T cell specificity was determined using MHCI tetramers and time-of-flight cytometry. Their results showed that TCR interactions have a clear competitive advantage, which manifests in the ablation of low-affinity antitumor T cell populations and reduction of therapy. The immunodominant population has a distinct phenotype profile that indirectly precludes the preexisting antitumor T cells from the site of the tumor. Further, a specific APL affinity of 0.25x relative to the OT-1 TCR expanded self-reactive T cells, allowing a balanced immune response against both antiviral and antitumor targets. The study suggests that the virus-infected tumor environment allows a balanced immune response. This means that while the immune system still recognizes and responds to the virus (retaining antiviral immunity), the engineered expression of altered TAAs helps focus T cells on attacking the tumor. This could increase the effectiveness of OV-based cancer treatments.

A technique for reprogramming tumors to express targetable antigens was also covered by Dr. Zaid Taha from the Ottawa Hospital Research Institute, Canada. Specifically, he described engineering a vesicular stomatitis virus (VSVΔ51) to cause cancer cells to exhibit a truncated, non-signaling HER2 antigen (HER2T). This antigen expression mimics “HER2+” status, enabling treatment with HER2-targeted therapies, like the antibody-drug conjugate trastuzumab emtansine (Kadcyla^®^). This approach showed synergistic tumor destruction in HER2-negative human tumor samples and mouse models. Moreover, combining VSVΔ51-HER2T with an oncolytic vaccinia virus encoding a HER2-targeted TCE further enhanced anti-tumor immune responses and efficacy across multiple tumor models. In order to precisely target tumors that were previously difficult to target, researchers also developed a dual-virus platform in which one virus introduces a target antigen to tumor cells and the other virus delivers a matching TCE. By providing new treatment options for a range of tumors, regardless of their unique antigen profiles, this strategy significantly increases the potential of currently available targeted therapies (21). In the United Kingdom, other research groups combined Ovs with other immunotherapies. Dr. Stephanie Drymiotou (The Francis Crick Institute, UK) presented data on using OVs in ovarian cancer. In their study, they engineered a recombinant virus lacking VGF and F1 (deltaVF) virus expressing NeonGreen (deltaVFTK-NG) to express Granulocyte-macrophage colony-stimulating factor (GM-CSF) for enhanced immune response and targeted tumor destruction in ovarian cancer. A high-throughput screen of 9,000 compounds was performed to identify drugs that enhance the ability of deltaVFTK-NG to induce murine ovarian cancer cell death. Three new recombinant viruses were created with deltaVF as the parental virus. Testing in human ovarian cancer cell lines and a mouse model of high-grade serous ovarian carcinoma showed that the combination of vinorelbine and deltaVFTK-NG-GM-CSF triggered ID8 Trp 53 -/- cell death by apoptosis and improved survival, with the virus selectively replicating in tumor sites. This research supports that Vinorelbine is a promising combination agent for deltaVFTK-NG-GM-CSF recombinant virus for the management of ovarian cancer (22). In another study highlighted by Amarin Wongariyapak (The Institute of Cancer Research London, UK) focused on the kinase-driven mechanisms of RP1 treatment. RP1 is an oncolytic herpes simplex virus developed for immunotherapy in patients with advanced solid tumors. The study assessed kinase activity and mapped phosphorylated amino acid sites using phosphoproteomics and high-throughput siRNA kinome screening. The results provided insight into important molecular pathways impacted by the virus by demonstrating dynamic changes in phosphoprotein levels over time in response to RP1. The siRNA screens identified kinase targets with potential for clinical application alongside RP1, showing their impact on cell death, viral spread, and Rel-A nuclear movement into the nucleus. These results underscore specific kinases as promising combination targets to boost RP1’s therapeutic impact. Further analysis is ongoing to validate these kinase candidates, aiming to deepen the understanding of RP1’s mechanisms of action.

These studies highlighted the game-changing potential of combining OVs with various immunotherapies to boost their effectiveness against tumors. They explored strategies like engineering OVs to reprogram the TME and trigger long-lasting immune responses, as well as identifying powerful synergies with targeted therapies.

## Clinical achievements

**Figure 2:**
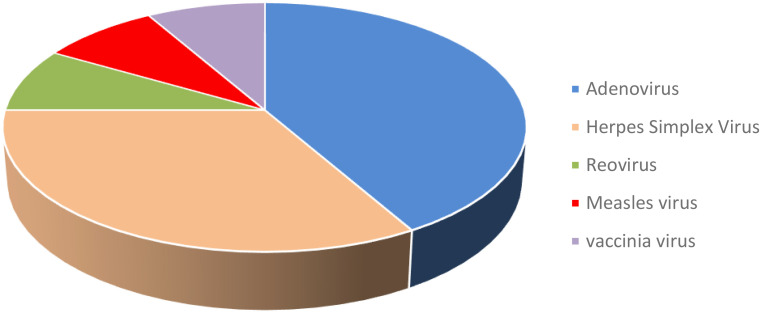
Summary of presented clinical trials with different types of OVs at the conference.

The meeting highlighted impressive data from recent clinical studies, including different OVs, which were shown to be effective across many cancer types (**Table 2**, **Figure 2**). Many studies highlight the promising potential of OAds as cancer therapeutics, showcasing their ability to elicit potent anti-tumor immune responses. In a study of Dr. Masato Yamamoto (University of Minnesota, USA), data on a novel OAd therapy for locally advanced PDAC, a highly aggressive cancer, was reported. The cGMP virus was purified using a double CsCl gradient, titrated, and vialed after being amplified using cGMP A549 using Cell Factory™. The engineered virus, RGDCRAdCOX2F, specifically targets PDAC cells while sparing normal tissues by using a COX-2 promoter, which activates the virus in cancerous tissue but remains inactive in healthy liver cells. Preclinical studies demonstrated that the virus replicates effectively within PDAC cells and causes cell death, with minimal toxicity in non-cancerous cells. Toxicological studies in animal models and tests on human tissue slices confirmed the virus’s safety and selectivity. With FDA approval for an investigational new drug (IND) application, the upcoming Phase I clinical trial will involve two patient cohorts: a non-resectable PDAC group to determine the maximum tolerated dose and a surgical group that will receive the virus before tumor resection.

**Table 2:** Summary of the clinical studies presented at the conference, including the used virus, cancer type, clinical phase, stage, and the clinical Trial.gov ID.

Virus Backbone	Virus Name	Cancer	Phase	Stage	ClinicalTrials.gov ID
Adenovirus	RGDCRAdCOX2F	Pancreatic Ductal Adenocarcinoma	Phase I	Ongoing	FDA IND-approved
	TILT-123	Various solid tumors, ovarian, lung, melanoma, head and neck	Phase I	Ongoing	NCT04695327, NCT04217473, NCT05271318, NCT05222932, NCT06125197
	ORCA-010	Prostate Cancer	Phase I/IIa	Ongoing	NCT04097002
	CAN-2409	Prostate Cancer	Phase II	Ongoing	NCT02768363
		PDAC	Phase II	Ongoing	NCR02446093
		NSCLC	Phase III/IS	Ongoing	NCT04495153
	DNX2401	Astrocytoma, Glioblastoma, Gliosarcoma	Phase I	Ongoing	NCT03896568
Herpes Simplex Virus	T-VEC	Advanced Melanoma	–	–	FDA-approved
	RP1	Advanced Melanoma, Solid Tumors	Phase III	Ongoing	NCT06264180
	CAN-3110	High-grade Glioma	Phase I	Ongoing	NCT03152318
	VG161	Hepatocellular Carcinoma	Phase I	Ongoing	NCT04806464
Reovirus	Pelareorep	Gastrointestinal Cancers (esp. mPDAC), Anal Cancer	Phase I/II	Ongoing	Eudra-CT: 2020-003996-16
Measles virus	MV-NIS	Medulloblastoma, ATRT Bladder Cancer	Phase I Phase I	Ongoing Ongoing	NCT02962167 NCT03171493
vaccinia virus Ankara	MQ710	Kaposi Sarcoma	Phase I	Ongoing	NCT05859074

In a study, Dr. Akseli Hemminki (University of Helsinki, Finland) presented data on the use of 10 OAds in 290 patients, highlighting a strong T-cell immune response, suggesting that anti-tumor immunity is a key mechanism. This insight led to the development of TILT-123 (igrelimogene litadenorepvec), a modified virus engineered to enhance T-cell activation by encoding TNFa and IL2. TILT-123’s capsid modifications support both intravenous and intratumoral administration. The mode of action of this virus is entry of the virus into cancer cells, destroying cancer cells, and producing more virus, attracting T cells, and stimulating their activity and finally supporting immune elimination of the tumor. Around 70 patients have received TILT-123 in five clinical trials (NCT04695327, NCT04217473, NCT05271318, NCT05222932, and NCT06125197), testing it as a monotherapy for solid tumors and in combination therapies with pembrolizumab (ovarian and lung cancer), avelumab (head and neck cancer, melanoma), and tumor-infiltrating lymphocytes (melanoma). TILT-123 has been safe in all trials to date (31.8.24), and there have been no dose-limiting toxicities in monotherapy or in combination with aPD-, aPDL1, or TILs. A Phase 1b trial with TILT-123, pembrolizumab, and liposomal doxorubicin for ovarian cancer is also ongoing (22). Also, Dr. Victor van Beusechem (Vrije Universiteit Amsterdam, Netherlands) showed data on immunological findings from a Phase I/IIa trial with oncolytic adenovirus ORCA-010 in treatment-naïve prostate cancer patients evidencing the development of tumour-specific immune response. Nine patients had a single intraprostatic injection as part of the trial, whereas twelve patients received two. Biopsies, prostatectomy samples, and blood were analyzed for immune cell population changes and tumor-specific T cells. According to the data, treating prostate cancer with an intraprostatic injection of ORCA-010 has shown encouraging outcomes. ORCA-010 induces immune activation in the TME, increasing cytotoxic T cells and systemic T cell activation, shifting the TME from cold to hot. Also, a systemic immune response against prostate-associated antigens induces and expands activated, proliferating memory phenotype CD8+ T cells in blood. The inspiring presentation, delivered by Dr. Robert Coffin (Replimune, USA), on T-VEC. He presented the first FDA-approved oncolytic virus therapy for advanced melanoma that he invented. He explained and demonstrated its efficacy in earlier stages of disease and in previously untreated patients, but also its limitations in advanced cases of melanoma. So, Coffin has been developing novel, more potent therapeutics belonging to Replimune’s novel oncolytic immunotherapy platform.

Coffin introduced a gene for GALV-GP-R in his vector and created a RP1 virus. The transduced gene codes for a glycoprotein that increases tumor antigen release to improve immune identification and stimulates highly ICD and fast cell killing via membrane fusion. Coffin also demonstrated RP1, an oncolytic virus combined with nivolumab to produce significant response rates and durability, even in cases of advanced disease with non-injected visceral lesions. Replimune’s novel oncolytic immunotherapy platform, which aims to increase tumor killing, intratumoral viral spread, and systemic immune activation, improving both local and distant tumor responses while enhancing the durability of therapeutic benefits, was the highlight of his presentation. Coffin presented promising data on RP1 combined with nivolumab, with an objective response rate (ORR) of 33.6% and median DOR of 21.6 months. He showed long-term therapeutic benefits and 1- (75.3%), 2- (63.3%), and 3-year (54.8%) 483 survival rates; the IGNYTE-3 confirmatory phase 3 trial 484 is presently enrolling (NCT06264180).

Another inspiring presentation was delivered by invited speaker, Dr. Antonio Chiocca (Brigham & Women’s Hospital, USA), who pointed out the clinical challenges and treatment timeline of GBM, mentioning the immunological coldness of GBM as the issue. Chiocca showed that the *in-situ* administration of OV leads to the transformation of the TME. He introduced CAN-3110, a genetically engineered oncolytic herpes simplex virus 1 (HSV-1), designed to enhance therapeutic efficacy and safety in recurrent high-grade glioma. Chiocca shared promising results from an ongoing clinical trial, demonstrating that *in-situ* administration of CAN-3110 transforms the TME and is well-tolerated. Chiocca demonstrated the well-tolerated longitudinal injections of CAN-3110 with serial biopsies, with a preliminary median overall survival (mOS) of 12.3 months with 3/6 patients alive. Chiocca highlighted that the serial biopsies are undergoing analysis through integrated multimodal “omics” to provide better understanding of tumor dynamics and therapeutic response (NCT03152318) (23).

One of the impressive presentations of the meeting was delivered by Dr. Francesca Barone (Candel Therapeutics, USA). Barone highlighted Candel’s pipeline, focusing on CAN-3110, a viral immunotherapy showing immune activation and tumor clearance linked to improved survival in a phase 1b trial, with results validated via paired biopsies and Intraoperative magnetic resonance imaging (MRI)-guided analysis. CAN-2409, their lead adenovirus-based therapy, delivers HSV-tk to tumors, converting valacyclovir into a cytotoxic metabolite to kill cancer cells and trigger systemic CD8+ T cell responses; it holds FDA Fast Track status for prostate cancer, PDAC, and Non-small cell lung cancer (NSCLC). Candel’s enLIGHTEN™ Discovery Platform uses HSV-based multi-gene payloads to reprogram the tumor microenvironment, addressing immunotherapy resistance through tailored, data-driven combinations (24).

Other clinical trial studies highlight the OVs potential in inducing immune responses, which can enhance the efficacy of treatments. Dr. Thomas C. Heineman (Oncolytics Biotech, USA) presented data on a GOBLET study, which is a Phase 1/2 clinical trial (Eudra-CT: 2020-003996-16) investigating the efficacy and safety of Pelareorep (pela), an intravenously administered reovirus, in combination with the PD-L1 inhibitor Atezolizumab (atezo) for advanced gastrointestinal cancers, specifically metastatic PDAC (mPDAC) and relapsed anal cancer. The study consists of three cohorts: 1) 1) newly diagnosed mPDAC patients receiving a combination of pela, atezo, and gem/nab; 2) newly diagnosed mPDAC patients randomized to receive pela + FOLFIRINOX with or without atezo; and 3) relapsed anal cancer patients treated with pela + atezo. Stage 1 results showed that the anal cancer cohort had an ORR of 37.5%, and evaluable mPDAC patients had an ORR of 62%, both of which exceeded predetermined success levels. According to these findings, pela is a novel immune-based cancer treatment that has strong efficacy signals against a variety of cancer types.

Another study, the PNOC005 Phase 1 clinical trial (NCT02962167), was presented by Dr. Sabine Mueller (University of California, USA and University Children’s Hospital Zurich, Switzerland). This trial assesses the safety and tolerability of intratumoral or intrathecal administration of the oncolytic measles virus (MV-NIS). It focuses on children and young adults with recurrent medulloblastoma (MB) or atypical teratoid/rhabdoid tumor (ATRT). Patients with localized recurrence (Stratum A) received MV-NIS directly into the tumor site during surgery, while those with disseminated recurrence (Stratum B) received it via lumbar puncture; a third group (Stratum C) received repeat doses for disseminated recurrent MB. Among 34 enrolled patients, 27 were evaluable for outcomes. The therapy was well tolerated, with only one dose-limiting toxicity and a few grades 3 or higher adverse events in medulloblastoma and pediatric ATRT. Post-MV treatment patient biospecimens revealed that MV-NIS sheds systemically after CNS delivery and naturally clears over time, and antiviral response after treatment is consistent with findings of acute viral infection. Blood analysis revealed immune activation patterns consistent with an antiviral response, with changes in B, T, and natural killer cells. MV-NIS treatment of immunocompetent preclinical models showed that it improved survival as monotherapy and upregulated gene pathways consistent with antiviral immunity. Therapy is safe and well-tolerated with minimal adverse effects. Immune markers and biologic correlates preliminarily indicated anti-viral effects in tumors (25).

Many other researchers explore the potential of OVs to treat cancers in immunocompromised patients, focusing on their ability to stimulate immune responses and enhance therapeutic outcomes. In Memorial Sloan Kettering Cancer Center in New York, researchers evaluate the safety, tolerability, and clinical efficacy of MQ710, a novel immune-activating viral platform, in patients with Kaposi sarcoma (KS). Dr. Liang Deng (Memorial Sloan Kettering Cancer Center, USA) indicated data on their study, which explores MQ710 (NCT05859074), an immune-activating, non-replicative viral therapy based on modified vaccinia virus Ankara (MVA), designed to treat KS, a vascular cancer linked to KS herpesvirus (KSHV) infection. Organ transplant patients who are experiencing chronic immune suppression, men who have sex with men, and people with HIV/AIDS are the groups most likely to get KS. Since there is currently no vaccination or accepted treatment for KS or KSHV, this experiment attempts to meet the pressing demand for efficient cures. MQ710 was engineered to delete the vaccinia immune evasion gene E5R and express immune-stimulating factors Flt3L and OX40L, enhancing antitumor immunity upon intratumoral delivery. In a Phase I clinical trial at Memorial Sloan Kettering Cancer Center, four HIV-negative KS patients received MQ710 IT injections: three at a lower dose and one at a higher dose, with treatment administered three times over nine weeks. Three patients achieved complete clinical and pathological responses, and one showed a partial response. MQ710 was well tolerated, with mild, transient flu-like symptoms reported. Early data indicate promising clinical responses and support further enrollment of KS patients, including those with HIV, to evaluate the broader efficacy of MQ710 in this challenging malignancy. Velia Penza (Mayo Clinic, USA) presented data on the immunological response and safety of MV-NIS, an oncolytic measles virus that targets bladder cancers’ Nectin4 and CD46 receptors, as a possible bladder cancer treatment. Over 90% of bladder cancer cases are urothelial carcinoma (UC), which present as non-muscle invasive bladder cancer (NMIBC) confined to the mucosa or muscle-invasive bladder cancer (MIBC). In their phase I trial (NCT03171493), 8 UC patients were recruited to receive 1x10^9^ TCID_50_ MV-NIS intravesically: 4 patients in the multi-dose expansion cohort received 2 doses, four and two weeks before radical cystectomy (RC), and 4 patients in the single-dose safety cohort received one dose four to one week before RC. To ascertain the pre- and post-treatment pathological stages, tumor specimens from pre-treatment transurethral resection of bladder tumor (TURBT) and post-treatment RC were examined. MV-NIS oncolytic virus indicated 100% safety and is well tolerated in all treated patients, with tumor downstaging observed in 50% of patients and a complete response (no residual disease) in three. MV infection was detected in 5/8 patients, and all patients showed significant immune infiltration. Tertiary lymphoid structures (TLSs) were present in all treated patients, and patients with pathological complete response showed a higher number of TLS formations. Dr. William Jia (Virogin Biotech Richmond, Canada) also presented data on a Phase I (NCT04806464) multicenter trial that evaluated VG161, an oncolytic HSV-1 virus, in HCC patients who have failed all standard therapies. The study involved intratumoral injections, guided by imaging, in different doses ranging from 1.0 × 10^8^ to 5 × 10^8^ PFU over 28-day cycles. VG161 showed great safety and promising efficacy (17.14%) in 35 late-stage HCC patients. Patients who received targeted therapies or ICI after treatment with VG161 experienced significantly longer survival compared to those who did not receive subsequent treatments. Overall survival was significantly influenced by independent factors, including prior ICI treatment lasting more than three months and subsequent anti-cancer therapies. Additionally, VG161 altered the TME with increased T/NK cells and myeloid cells, which occurred not only in the injected tumor but may also have happened in the abscopal tumors.

To explore if OV as immunotherapy relies on the hypothesis that viral oncolysis induces inflammatory tumor cell death, recruiting APCs to the tumor, releasing TAAs, and facilitating their presentation to T cells to generate systemic anti-tumor immunity, researchers at Mayo Clinic, presented by Dr. Richard Vile, utilized a slow-developing model of HCC in mice, induced by the integration of ß-catenin and hMet oncogenes via a Sleeping Beauty transposon system (SB-HCC). Tumor-bearing mice showed partial responsiveness (~50% long-term cures) to anti-PD-L1s immune ICIs, mimicking current clinical outcomes (NCT01628640). However, the addition of VSV-IFNß to anti-PD-L1 therapy unexpectedly abolished its therapeutic effects by promoting dominant anti-viral CD8+ T cell responses and suppressing anti-tumor T cell populations targeted by ICIs. These changes were marked by alterations in immune markers such as CD38, CD39, PD-L1, PD-1, Granzyme B, and CD44. To counter this, a cDNA library derived from SB-HCC tumors was used to encode tumor antigens within VSV, redirecting a portion of the anti-viral T cell response towards anti-tumor activity. This approach significantly enhanced therapeutic outcomes, even without additional anti-PD-L1 therapy. These findings highlighted the potential pitfalls of highly immunogenic viruses in OV, which may overshadow weak anti-tumor T cell responses, but also underscore the promise of incorporating tumor antigens into OVs to effectively chimerize anti-viral and anti-tumor T cell responses, paving the way for improved immune-based tumor clearance and enhanced synergy with ICIs. Dr. Vile also presented another study on oncolytic virotherapy as T cell immunotherapy. Researchers developed an oncolytic vesicular stomatitis virus expressing IFNß (VSV-IFNß) that showed initial clinical promise but faced tumor escape driven by a dominant-negative CSDE1C-T mutation identified in resistant tumors. By co-evolving the virus to adapt to this mutation, they created a novel escape-adapted virus (VSV-IFNß-IGR-P/MC-U) with significantly enhanced replication in resistant cells. Additionally, introducing wild-type CSDE1 expression into VSV (VSV-IFNß-CSDE1WT) improved viral replication and reduced escape. Combining VSV-IFNß-CSDE1WT with immune checkpoint blockade therapy, optimized for late treatment, demonstrated superior anti-tumor efficacy. A Phase I/II trial in dogs with melanoma revealed safety, viral replication, and preliminary immune responses. These findings pave the way for future trials using CSDE1-enhanced and escape-targeting VSVs in both canine and human patients (26).

## Young investigators talks

This year, a new section titled “Young Investigators Talks” was introduced, where emerging researchers from all over the world presented their latest data on oncolytic virotherapy. The topics covered preclinical advancements, the TME, novel platforms and payloads, mechanisms of action, biomarkers of response, and clinical trials targeting various cancers. In order to improve the oncolytic potential of viruses as well as the anticancer immune responses they elicit, a large number of studies focused on the TME. Dr. Marjan van de Merbel (Doctor) (Leiden University Medical Center, Netherlands) presented a study investigating the effects of OVs as a potential therapeutic approach for prostate cancer. They developed an *in vitro* model that could accurately mimic the interactions between cancer cells and immune cells. Using a 3D co-culture system of prostate cancer tumoroids and immune cells, they examined the effects of two promising OVs, Jin-3 and Goravir, on cancer cell death and immune activation. *In vitro* results demonstrated that viral exposure led to direct tumor cell killing and immune stimulation, resulting in reduced numbers of viable prostate cancer cells. They also observed augmented release of fragmented keratin 18 release (indicative of prostate cancer apoptosis) and CXCL10 and IFNgamma production (indicative of chemoattraction of immune effector cells and ICD). This 3D tumoroid-immune cell co-culture represented a valuable model to test both direct and indirect effects of oncolytic virotherapy, and Jin-3 and Goravir showed distinct responses within this model. This study is currently ongoing to address the effect of OVs-immune ICI combinations. Another study, presented by Dr. Maria Davola (McMaster University, Canada), explored the potential of bovine herpesvirus type 1 (BHV-1) as a treatment for PDAC. Using a humanized mouse model of PDAC that more closely resembles human immune responses, they sought to determine if BHV-1 could successfully inhibit tumor growth by inducing an immune response within the tumor environment. Given BHV-1’s ability to attract CD8+ tumor-infiltrating lymphocytes (TILs), they tested its impact both alone and in combination with other immunotherapies. Results showed that BHV-1 significantly reduced tumor growth in humanized models and increased levels of B cells, CD4+, and CD8+ T cells in responding animals, while it showed no effect in highly immunodeficient mice. This study underscores BHV-1’s potential as a promising immunotherapy for PDAC and opens up new ideas for understanding how it works in the immune system.

Other group researchers’ goal was to enhance the effectiveness of Reo therapy in pancreatic tumors by targeting cancer-associated fibroblasts (CAFs). Nicole Dam (Leiden University, Netherlands) presented their study on using a genome-wide CRISPR/Cas9 screen to identify genes regulating JAM-A expression on fibroblasts, which can be targeted to sensitize CAFs to reovirus treatment. F11R, the gene encoding JAM-A, was identified as the top positive regulator of JAM-A expression. The top negative regulators identified were Fibroblast Growth Factor Receptor 1 (FGFR1) and Zinc Finger E-box Binding Homeobox 1 (Zeb1). Zeb1 inhibition can sensitize CAFs to reovirus-induced cell death, providing a rationale for combining Zeb1 inhibitory drugs with Reo treatment to improve the killing of CAFs and boost overall tumor eradication.

In order to improve OVs’ safety and effectiveness, other groups worked to create and refine OVs for cancer treatment. By either improving the virus’s replication in tumor cells or adding genes that boost immune response and virus spread, these groups attempted to improve the virus so that it more effectively targets cancer cells. In a study presented by Anne Everts (Erasmus MC, Netherlands), comparing the safety and direct oncolytic efficacy of intravenous injections with two lentogenic NDV variants in orthotopic pancreatic cancer-bearing mice for 50 days, the study showed that both NDV F0 and NDV F0-M/S were safe and highly effective in treating orthotopic PDAC. The mice received injections of NDV F0, NDV F0-M/S, or PBS after having 2.5E+05 PDAC cells implanted in their pancreas. The findings demonstrated that mice treated with NDV F0 exhibited significantly higher levels of antiviral cytokine expression than mice treated with NDV F0-M/S. Additionally, there were no discernible differences between the two groups treated with NDV variants, and both groups exhibited a significant decrease in tumor volumes for 21 days after the initial treatment. The study suggests that the differences in early cytokine responses between animals treated with the two viruses may be correlated to differences in antitumor immune responses. In another study, Dr. Taha Azad (Sherbrooke University, Canada) reported an iterative strategy exploiting viruses’ susceptibility to certain antibiotics, which has been used to accelerate genome engineering and identify dispensable genes for replication. This approach has been applied for insertional mutagenesis, creating libraries of HSV-1 and Vaccinia virus that express transgenes for immunomodulatory cytokines and identify stable transgene-insertion sites and gene deletions to enhance oncolytic virus safety and effectiveness. This study provides a promising method for advancing the development of genetically modified OVs, potentially to improve their therapeutic potential in cancer treatment.

Research on creating a controllable system for expressing therapeutic proteins in OAds using RNA-switch technology was presented by Laura Kayser of the German Cancer Research Center (DKFZ). The aim was to optimize oncolytic virotherapy by allowing external control over immune-stimulatory protein expression, improving treatment effectiveness, and ensuring safety. Kayser’s team evaluated various tetracycline-responsive RNA-ON switches and successfully constructed OAds that induced luciferase expression up to 26 times upon Tet addition. With expression levels exceeding 100-fold, the most efficient RNA switch was then modified to regulate the expression of therapeutic cytokines, such as IL-2, IL-12, and IFNβ. This study highlights the broad applicability of RNA switches in gene and virotherapy, as well as their capacity to control gene expression independently of the transgene. Future studies aim to explore the controlled delivery of cytokines *in vivo* to enhance anti-tumor immunity while serving as a safety mechanism in case of adverse effects.

Other researchers’ goal was to evaluate the potential of coxsackievirus A21 (CVA21) as an oncolytic immunotherapy for Ewing sarcoma (ES), a rare and aggressive pediatric cancer. ES is a rare pediatric cancer with poor prognosis despite chemoradiotherapy and surgery. Coxsackievirus A-21 (CVA21) is an emerging immunotherapy with potential for targeted therapies in ES. CVA21 preferentially replicates in cancer cells due to overexpression of the viral entry receptor, intercellular adhesion molecule (ICAM)-1. A study presented by Dr. Richard Baugh (University of Leeds, UK) performed using patient-derived ES (PDES) cells showed that all PDES cultures expressing ICAM-1 were sensitive to CVA21-mediated oncolysis even at low doses. Pre-treatment with inducers of ICAM-1 expression, such as TNF-α and ATRA, increased ICAM-1 expression and significantly enhanced CVA21-mediated oncolysis. CVA21 is a promising oncolytic candidate for ES due to high expression of the viral entry receptor, ICAM1.

There was also a study that explored the potential of oncolytic virotherapy using T-VEC combined with the immune ICI pembrolizumab for treating NUT carcinoma (NC), a rare and aggressive tumor with poor prognosis. This study, presented by Dr. med. Linus Kloker from the University of Tübingen, Germany, found that T-VEC was well-tolerated and showed promising effects, including reduced oxygen supply to tumors, partial treatment responses, and increased infiltration of CD8+ T cells within the tumor. A Phase I/II investigator-initiated trial is now being planned for both pediatric and adult patients with neuroendocrine carcinoma (NC), focusing on safety and feasibility. The primary goal of the trial is to assess safety and feasibility, while exploratory endpoints will include early signs of efficacy, virus replication, T-cell responses to tumor-specific peptides, and patient-reported outcomes.

During the conference and in addition to the outstanding talks, the reported studies were also displayed in 68 posters, including three posters on clinical trials, 19 posters on combination therapies, 23 posters on novel platforms and payloads, 12 posters on mechanisms of action, seven posters on tumor microenvironment, and four posters on biomarkers of response. Our authors presented for the first time a poster under the combination therapies section which reported that microglia cells infected with VV, trigger a response that significantly impairs the viability and survival of neuroblastoma cells due to virus replication and oncolysis.

## Golden virus award and final keynote

The Golden Virus Award was given to one exceptional OV researcher, as is now customary at IOVC. Dr. Antonio Chiocca gave Dr. Juan Fueyo the award this year in honor of his longstanding leadership in the OV area and contributions to it. Dr. Juan Feuyo serves as a Neuro-Oncologist at MD Anderson Cancer Center, USA. His research primarily focuses on the development of novel gene therapy strategies for the treatment of malignant gliomas. His group developed Delta-24-RGD and advanced it through preclinical testing into clinical trials. Dr. Juan Feuyo kindly asked his colleagues and his wife, Dr. Candelaria Gomez-Manzano to join him when he was getting the Golden Virus Award. A closing keynote talk delivered also by Dr. Juan Feuyo on the Viroimmunotherapy for Gliomas. Feuyo introduced oncolytic adenovirus Delta-24-RGD, as one of the most efficient Ovs as it has a 24 bp deletion for cancer selective replication and insertion of RGD-4C, enhances the infectivity of virus. A phase I clinical trial of Delta-24-RGD (DNX-2401) showed antiglioma efficacy in 37 patients with recurrent malignant gliomas. Results showed a 95% tumor reduction in group A patients, 20% survival for over three years, and progression-free survival in group B. The study suggests that some patients’ clinical reactions are influenced by an active immunological response (27). Another clinical study evaluated the safety and adverse-event profile of oncolytic viral therapy (DNX-2401) in pediatric patients with diffuse intrinsic pontine glioma. Adverse events included headache, nausea, vomiting, fatigue, and hemoparesis. Some patients experienced tumor reduction, partial response, or stable disease. Feuyo also indicated clinical translation of VCN-01 virus (designed and generated by Dr. Alemany), resulting in tumor-selective replication, anti-tumor activity and immune activation. Viral infection attracts lymphocytes to tumor sites, leading to viral elimination. However, tumors may remain cold for anti-tumor responses. A multicenter study evaluated the combination of oncolytic virus DNX-2401 and pembrolizumab in recurrence Szarent glioblastoma, with a safe and well-tolerated combination, with 56.2% of patients having stable disease. The presentation highlighted the need for balance in viral immunogenicity, immunodominance, and tumor-specific immunity strategies. Combining OVs with immunotherapy shows promise but requires optimization to mitigate enhanced anti-viral responses. Pseudoprogression suggests durable responses. Additionally, modifying the microbiome and leveraging molecular mimicry between viral and tumor antigens could further enhance anti-tumor effects. Together, these insights guide the rational design of oncolytic virus therapies to maximize their clinical potential.

The take home message of the conference is that the immunodominance of the anti-viral response over the anti-tumor response may underlie the long-term failure of oncolytic virotherapy and it should be proposed an approach to redirect this, as emphasized in many presentations.

## Conclusion

Compared to the previous conference, the meeting highlighted that OV therapy for cancer is progressing well. However, it remains difficult to compare the efficacy of different types of OVs due to variations in replication, survival in different host tissues, and other differences. At this time, one cannot make a definitive choice between different routes of application, e.g. intravenous or intertumoral delivery of the viruses. Notably, the immune response to different cancers may play an important role when combined with viruses or when delivering viruses using engineered stem cells. The authors believe that autologous cell-mediated OV therapy may help overcome the current challenges.

At the end, we would like to add that the date and host city for the follow-up meeting (next year) have not yet been disclosed.
